# Donor-Derived Cell-Free DNA at Diagnosis of Cardiac Allograft Rejection Stratifies Risk of Mortality and Graft Dysfunction

**DOI:** 10.1161/CIRCHEARTFAILURE.125.013718

**Published:** 2026-06-15

**Authors:** Zaid N. Safiullah, Han Su, Hyesik Kong, Moon Kyoo Jang, Keyur Shah, Gerald J. Berry, Palak Shah, Hannah A. Valantine, Xin Tian, Sean Agbor-Enoh

**Affiliations:** Critical Care Medicine Department, National Institutes of Health, Bethesda, MD (Z.N.S.).; Genomic Research Alliance for Transplantation (GRAfT), Bethesda, MD (Z.N.S., H.K., M.K.J., K.S., G.J.B., P.S., H.A.V., S.A.-E.).; Laboratory of Applied Precision Omics (APO), National Heart, Lung, and Blood Institute, National Institutes of Health, Bethesda, MD (Z.N.S., H.K., M.K.J., S.A.-E.).; Office of Biostatistics Research, National Heart, Lung, and Blood Institute, National Institutes of Health, Bethesda, MD (H.S., X.T.).; Stanford University School of Medicine, Stanford, CA (G.J.B., H.A.V.).; Department of Heart Failure and Transplantation, Inova Heart and Vascular Institute, Falls Church, VA (P.S.).; Virginia Commonwealth University, Richmond, VA (K.S.).; George Washington University, Washington, DC (H.S.).

**Keywords:** cell-free nucleic acids, biomarkers, graft rejection, heart transplantation

## Abstract

**BACKGROUND:**

Cardiac acute rejection (AR) is a risk factor for poor outcomes; however, there are limited risk prediction models to stratify these patients for death or prolonged left ventricular (LV) dysfunction. We assessed the prognostic utility of percent donor-derived cell-free DNA (%dd-cfDNA) measured at AR diagnosis for predicting adverse outcomes.

**METHODS:**

The prospective multicenter GRAfT study (Genomic Research Alliance for Transplantation) enrolled heart transplant recipients and collected serial plasma samples to quantitate %dd-cfDNA. AR was defined as acute cellular rejection, antibody-mediated rejection, as well as biopsy-negative antibody-mediated rejection (donor-specific antibody positivity with LV dysfunction). In the primary analyses, AR was stratified by %dd-cfDNA at diagnosis using a data-driven threshold of 0.15%. Cox regression models assessed the associations between the time-dependent covariates of AR and %dd-cfDNA levels at the AR diagnosis and the outcome of prolonged LV ejection fraction decline (≤50% for ≥90 days) and death.

**RESULTS:**

The study included 277 patients and 3218 %dd-cfDNA measurements. Over a median follow-up of 4.9 years (interquartile range, 2.5–5.0), 53 patients experienced the composite outcome of death or prolonged LV dysfunction, and 75 (27%) patients developed AR, including 43 (15%) patients with acute cellular rejection, 18 (7%) with pathological antibody-mediated rejection, and 14 (5%) with donor-specific antibody+/LV dysfunction. AR was associated with an increased risk of the primary composite outcome (hazard ratio, 4.47 [95% CI, 2.42–8.26]; *P*<0.001). When AR was stratified by %dd-cfDNA at diagnosis, patients with %dd-cfDNA ≥0.15% had higher risks of prolonged LV dysfunction, death, and the composite outcome compared with patients who had not developed AR at the same follow-up time (hazard ratio, 6.28 [95% CI, 3.04–13.0]; *P*<0.001 for the composite outcome). In contrast, the risks of death and prolonged LV ejection fraction reduction were not statistically significantly increased among patients who developed AR with %dd-cfDNA <0.15% at diagnosis.

**CONCLUSIONS:**

AR with elevated %dd-cfDNA levels at diagnosis is associated with an increased risk of adverse outcomes after heart transplant, offering novel prognostic utility.

**What is New?**

- In this multicenter study, percent donor-derived cell-free DNA (%dd-cfDNA) at acute rejection diagnosis stratified cardiac transplant patients by risk of death and prolonged left ventricular dysfunction.
- Patients with %dd-cfDNA ≥0.15% at rejection diagnosis had a 6-fold higher risk of dysfunction and death than those with <0.15%.
- Furthermore, high-risk patients had persistently elevated %dd-cfDNA after antirejection treatment compared with low-risk patients, supporting %dd-cfDNA as a biomarker of treatment response.

**What Are the Clinical Implications?**

- %dd-cfDNA is noninvasive, available clinically, and represents a bedside tool to stratify patients for adverse outcome risk. %dd-cfDNA levels ≥0.15% at the time of rejection diagnosis may warrant intensified monitoring or therapy escalation.
- %dd-cfDNA trends after antirejection treatment may represent a biomarker to identify poor treatment response. These candidates may benefit from second-line immunosuppression or prolonged treatment.
- Together, these findings support biomarker-driven, personalized treatments in cardiac transplantation.

Acute rejection (AR) continues to be a significant cause of morbidity and a risk factor for mortality in the heart transplant recipients despite advances in immunosuppressive therapies.^1^ The estimated incidence of AR at 2 years post-transplant in a large multicenter cohort is 62%.^2^ Acute cellular rejection (ACR) is a common posttransplant complication, with an incidence of 10% to 14%.^3,4^ The incidence of antibody-mediated rejection (AMR) is variable with a wide range of 3% to 85%, likely due in part to diagnostic limitations.^5,6^ There is currently no available tool in clinical use to identify patients with AR who are at increased risk of poor long-term outcomes.

In the current era of cardiac transplantation, with more effective treatment protocols and updated definitions, the mortality and morbidity associated with AR are not well understood. In prior studies, increasing severity of ISHLT (International Society for Heart and Lung Transplantation) histological grade in AR and recurrent episodes were associated with increased mortality, development of coronary artery vasculopathy, and retransplantation.^7–9^ These sometimes decades-old studies that established these associations were often single-center, retrospective, and did not use more contemporary definitions of AMR or include endomyocardial biopsy (EMB)-negative forms of AMR,^10–12^ which are important risk factors for mortality^13^ and coronary artery vasculopathy.^14,15^ The studies also relied on EMB for grading AR severity, an invasive approach limited by a high interrater variability to grade AR and a low diagnostic yield, even when patients presented with significant allograft dysfunction.^16^ Noninvasive biomarkers, such as percent donor-derived cell-free DNA (%dd-cfDNA), now present sensitive alternatives to assess allograft injury and severity.^1,17,18^ Elevated levels of %dd-cfDNA are associated with both contemporarily defined ACR, AMR, and subclinical injury, often preceding histological changes detectable by EMB,^18^ as well as with EMB-negative AMR.^13^ Percent dd-cfDNA could, therefore, capture severe forms of AR associated with important clinical outcomes.

The lack of reliable clinical end points also limits risk stratification in heart transplantation. While mortality remains the most definitive adverse outcome, its low incidence limits clinical trial design. Significant and prolonged reductions in left ventricular (LV) function represent a critical intermediate end points that strongly correlate with morbidity, quality of life, and long-term graft survival, even in the absence of high-grade histological rejection.^12^ Historically, both ACR and AMR are associated with echocardiographic allograft dysfunction.^10,11,19^ A composite end point of prolonged reduction in LV ejection fraction (LVEF) and mortality could therefore provide an intermediate- and long-term benchmark to assess the risk of AR.

This multicenter prospective cohort study aim to evaluate the prognostic significance of %dd-cfDNA levels measured at the time of AR diagnosis and to investigate the association between AR and mortality and morbidity. We hypothesize that elevated %dd-cfDNA levels are associated with an increased risk of mortality and prolonged reduction in LVEF, and we posit that AR is associated with mortality and prolonged allograft dysfunction even in the current era with advances in treatment strategies. By elucidating the prognostic utility of %dd-cfDNA, our goal was to enhance risk stratification and guide personalized management strategies for patients with AR.

## Methods

### Study Design

The GRAfT study (Genomic Research Alliance for Transplantation, REGISTRATION: URL: https://www.clinicaltrials.gov; Unique identifier: NCT02423070) enrolled heart transplant recipients aged ≥18 years who were on the waitlist before the transplant and followed them serially after the transplant. Patients undergoing repeat or multiorgan heart transplantation were excluded. Patients were recruited at 5 regional transplant centers: Inova Schar Heart and Vascular, The Johns Hopkins Hospital, the University of Maryland Medical Center, Virginia Commonwealth University, and MedStar Washington Hospital Center. The full study methodology, including center immunosuppression and surveillance protocols, has been previously published.^18^ We collected data between 2015 and 2025. The institutional review boards of all centers and the National Heart, Lung, and Blood Institute approved the study, and patients provided their informed consent before enrollment. This study adheres to the principles of the Declaration of Helsinki and the ISHLT statement on Transplant Ethics. The data that support the findings of this study are available from the corresponding author upon reasonable request.

### Posttransplant Evaluations

Patients underwent surveillance and clinically indicated EMB and other evaluations, as part of routine clinical care. The individual center’s pathologist used the ISHLT grading system to grade for AMR and ACR. We defined AMR as pathological AMR (pAMR) 1 (H+), 1 (I+), 2, or 3. We also included a recent category synonymous with EMB-negative AMR defined as a positive donor-specific antibody (DSA) evaluation with LV dysfunction, defined as an LVEF decrease by ≥10% from the previous measurement to an LVEF ≤50%.^13^ Luminex (Luminex, Austin, TX) multiplex bead assays were used to evaluate the presence, phenotype, and quantity of antibody-bead binding of DSA, with a mean fluorescence intensity threshold of 1000 used to determine a positive DSA evaluation. We defined mild-to-moderate AR as ACR grade 2 or pAMR grade 1, and severe AR, defined as ACR grade 3, pAMR grade ≥2, or DSA+LV dysfunction. We defined control subjects as those without pAMR+, ACR+, or DSA-positive findings with LV dysfunction.

### Outcome Measures

The primary composite outcome was death or prolonged LV dysfunction (≤50% for ≥90 days). The secondary outcomes included all-cause death and prolonged LV dysfunction occurring during the posttransplant follow-up period. Given the paucity of events of prolonged LV dysfunction, analyses for this endpoint were considered exploratory.

### Percent dd-cfDNA Measurement

We genotyped the donor and recipient DNA to identify informative single-nucleotide polymorphisms. After transplantation, we isolated plasma cfDNA for library construction and paired-end shotgun sequencing. Sequence reads were analyzed to identify donor, and recipient reads using informative single-nucleotide polymorphisms. We computed %dd-cfDNA as a percentage of reads with donor single-nucleotide polymorphisms to reads of donor plus recipient single-nucleotide polymorphisms.^18,20^ Given known early posttransplant exponential decay,^21^ %dd-cfDNA measurements <30 days posttransplant were excluded. In the primary analyses, elevated %dd-cfDNA was set as a data-driven threshold ≥0.15%, which selected via grid search in our cohort; and an alternative threshold ≥0.25% as measurement by a previous study,^18^ was evaluated in sensitivity analysis.

### Statistical Methods

Descriptive statistics were used to summarize baseline recipient and transplant characteristics. Continuous variables are reported as medians (interquartile range), and categorical variables as counts and percentages. Cumulative incidences of pAMR, ACR, or DSA+/LV dysfunction were estimated using competing-risk methods, with death before the event of interest treated as a competing-risk event. Kaplan-Meier curves were used to describe the probabilities of survival and the composite outcome of prolonged LV dysfunction and death; patients were censored at the last follow-up, and no earlier deaths were excluded from the analyses. For each patient, only AR episodes occurring ≥30 days after transplant were included, with perioperative AR (<30 days) excluded. Levels of %dd-cfDNA at the diagnosis of AR were log10-transformed and compared with those of control patients without AR (AR−). Measurements of %dd-cfDNA within 30 days after transplantation were excluded to avoid perioperative variability; subsequent measurements (≥30 days) were modeled as time-dependent exposures, while time-to-event outcomes (including earlier death) were analyzed from the time of transplant. Although %dd-cfDNA was measured longitudinally, we focused on values at the time of AR diagnosis; pre-AR prediction was not assessed due to irregular sampling. Each AR case was time-matched (±1 day) in a 1:2 ratio to AR- controls. Differences in the mean %dd-cfDNA were assessed using a linear mixed model that included patient subgroup as a fixed effect and a subject-specific random intercept to account for repeated measures. To examine the association with prolonged LV dysfunction and death, univariate and multivariable Cox regression analyses were performed, with the first AR episode included as a time-dependent covariate. Time since transplant was used as the time scale for the time-to-event analyses. Furthermore, we assessed the associations between transplant outcomes and AR dichotomized by %dd-cfDNA at diagnosis (≥0.15% versus <0.15%). This data-driven cutoff of 0.15% was selected via grid search to optimize model performance. The incremental predictive value of adding %dd-cfDNA categorization to the AR-only model was evaluated using net reclassification improvement and integrated discrimination improvement, which reflect improvements in risk reclassification and discrimination, respectively; positive net reclassification improvement/integrated discrimination improvement values indicate improved prediction with %dd-cfDNA. In sensitivity analyses, we also explored models with AR stratified by different %dd-cfDNA thresholds and by both severity and %dd-cfDNA, as well as the trend of %dd-cfDNA after AR diagnosis. Hazard ratios (HRs) and 95% CIs were reported. All tests were 2-sided, with *P*<0.05 denoting statistical significance. Statistical analyses were conducted using R statistical software (version 4.5.0; R Foundation for Statistical Computing).

## Results

### Patient Population

**Figure 1.**
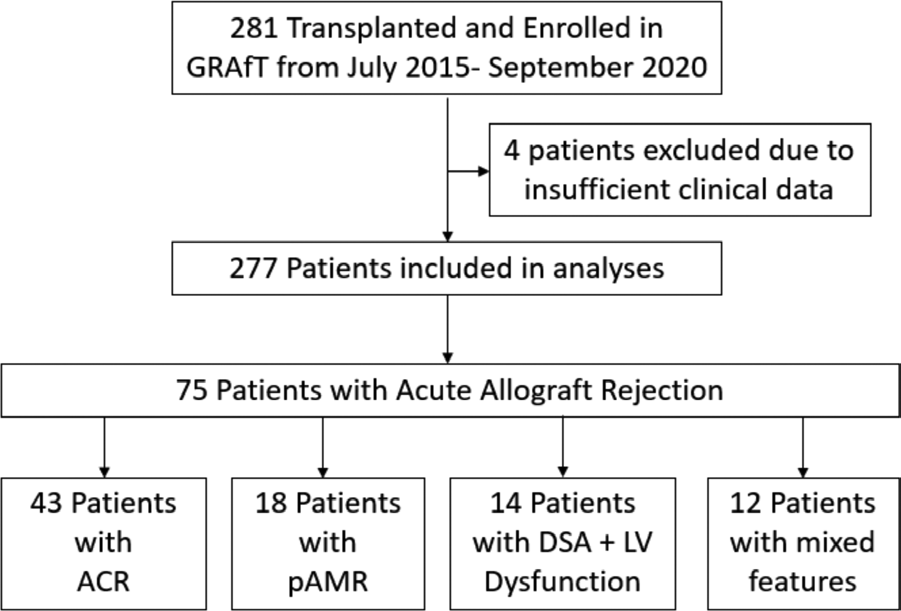
**Flowchart of study participants.** Mixed features indicate overlap across rejection phenotypes; not an additional mutually exclusive category. ACR indicates acute cellular rejection; DSA, donor specific antibodies; GRAFT, Genomic Research Alliance for Transplantation; LV, left ventricular; and pAMR, pathologic antibody mediated rejection.

The study enrolled 281 heart transplant recipients between July 2015 and September 2020. After excluding 4 patients with insufficient clinical data, 277 patients were included in the analysis (Figure 1). A total of 3218 %dd-cfDNA measurements were obtained, with a median of 12 measurements per patient (interquartile range, 7–16; Figure S1). The median age was 56 years (interquartile range, 48–62); 74% were men, 47% were White, and 57% of patients underwent transplantation for a nonischemic cardiomyopathy. Baseline characteristics for the overall cohort and by AR status are summarized in Table 1.

**Table 1.**
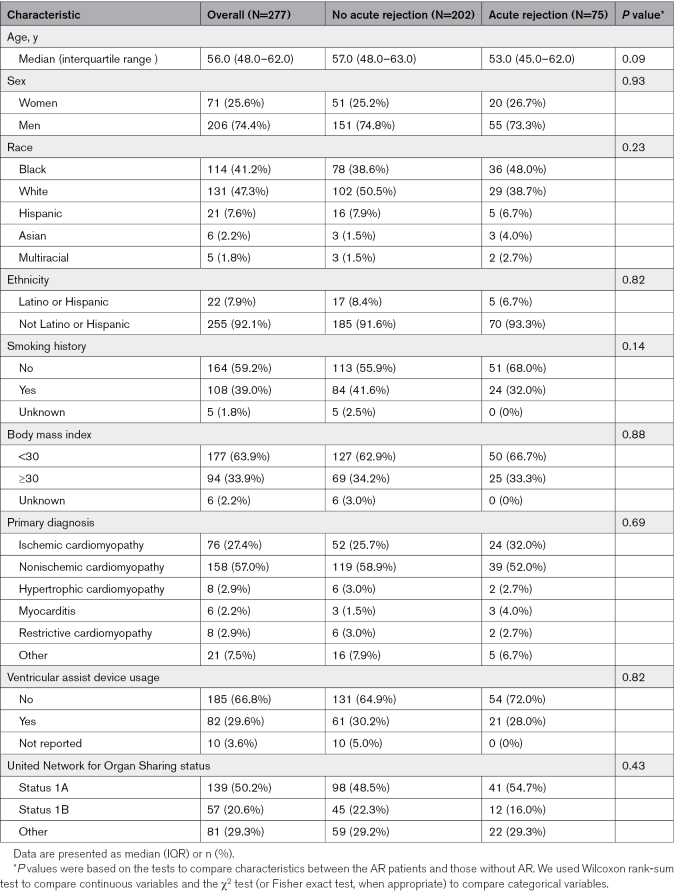
Baseline Patient and Transplant Characteristics

**Figure 2.**
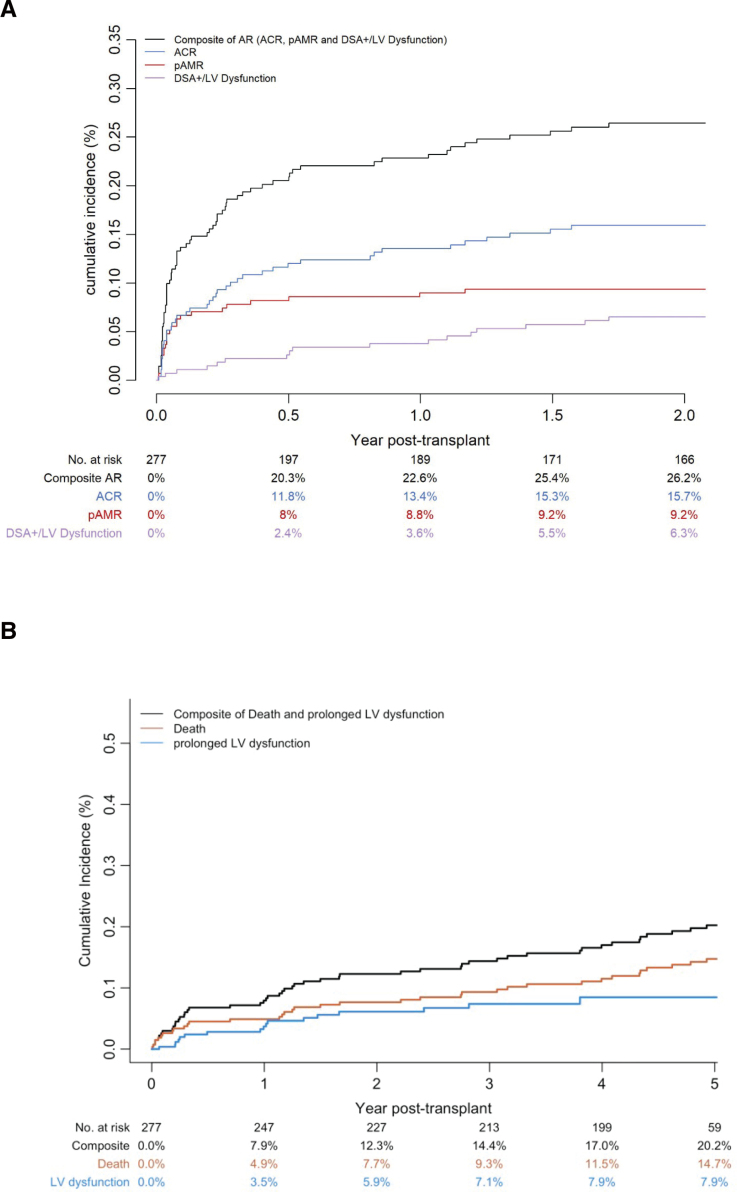
**Cumulative incidence of acute rejection (AR) phenotypes and clinical outcomes. A**, Cumulative incidence of AR phenotypes, including ACR, pAMR, AMR by DSA+/LV dysfunction and the composite of these events. **B**, Cumulative Incidence of Clinical Outcomes. The composite outcome includes the prolonged LV dysfunction or death. The composite cumulative incidence curve (black) represents the time to the first event of its individual components. ACR indicates acute cellular rejection; DSA, donor specific antibodies; LV, left ventricular; and pAMR, pathologic antibody mediated rejection.

The distribution of AR phenotypes is shown in Figure 1. Overall, 75 of 277 patients (27%) developed AR, including 43 of 277 (15%) with ACR, 18 of 277 (7%) with pAMR, and 14 of 277 (5%) with DSA+/LV dysfunction.Figure 2A shows the cumulative incidence of AR categories (with earlier death without AR treated as a competing event): the 2-year cumulative incidences were 15.7% for ACR (95% CI, 11.5–20.4%), 9.2% for AMR (95% CI, 5.9–12.9%), and 6.3% for DSA+/LV dysfunction (95% CI, 3.5–9.5%), respectively. Among the 75 patients with AR, 32 of 75 (43%) experienced severe AR (64 episodes), and 43 of 75 (57%) had mild-to-moderate AR (79 episodes).

The cumulative incidence of the primary composite outcome (death and prolonged LV dysfunction ≤50% sustained for ≥90 days) and its individual components is shown in Figure 2B. At the 5-year follow-up, the cumulative incidence was 20.2% for the composite end point, 14.7% for death, and 7.9% for prolonged LV dysfunction (death without prolonged LV dysfunction was treated as a competing event). The composite end point was driven primarily by mortality: 12% died without prior prolonged LV dysfunction, 6% had prolonged LV dysfunction without subsequent death, and 2% experienced prolonged LV dysfunction followed by death.

### AR Is Associated With Poor Outcomes

AR was associated with an increased risk of the composite primary outcome of death or prolonged LV dysfunction in both univariate analysis (HR, 4.48 [95% CI, 2.43–8.27]; *P*<0.001) and multivariable models adjusted for age, sex, and race (adjusted HR, 4.47 [95% CI, 2.42−8.26], *P*<0.001; Table 2). In multivariable analysis, the association remained significant for the different phenotypes of AR, including AMR defined by biopsy or DSA+/LV dysfunction (HR, 3.10 [95% CI, 1.51–6.39]; *P*=0.002), and ACR (HR, 4.97 [95% CI, 2.48–9.94], *P*<0.001; Table 2). AR and its phenotypes were also associated with an increased risk of death (Table 2). Similarly, AR and ACR were associated with an increased risk of prolonged LV dysfunction (Table 2). When AR was stratified by severity (severe or mild-to-moderate) and adjusted for patient’s age, sex, and race, severe AR was associated with a higher risk of the composite outcome (HR, 6.30 [95% CI, 2.91–13.6]; *P*<0.001) and death (HR, 4.82 [95% CI, 2.00–11.6], *P*<0.001; Table S1). Severe AR defined by biopsy alone also showed an increased risk of poor outcomes (Table S1). Mild-to-moderate AR was associated with the composite outcome (HR, 3.11 [95% CI, 1.40–6.90]; *P*=0.005) but was not associated with death (Table S1).

**Table 2.**
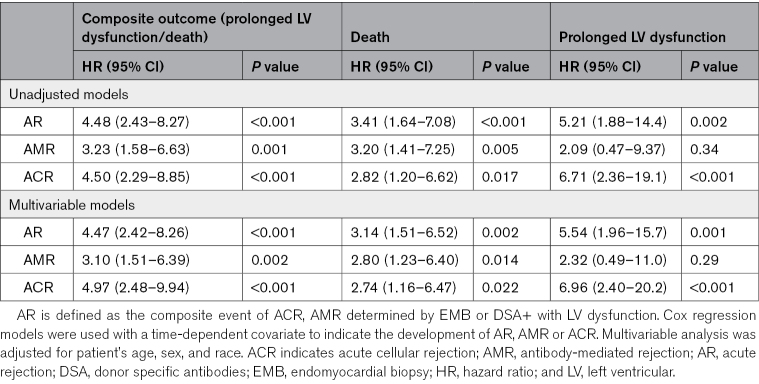
Association of Acute Rejection With the Risk of Death and Prolonged LV Dysfunction

### Elevated %dd-cfDNA Is Associated With Adverse Outcomes in AR

**Figure 3.**
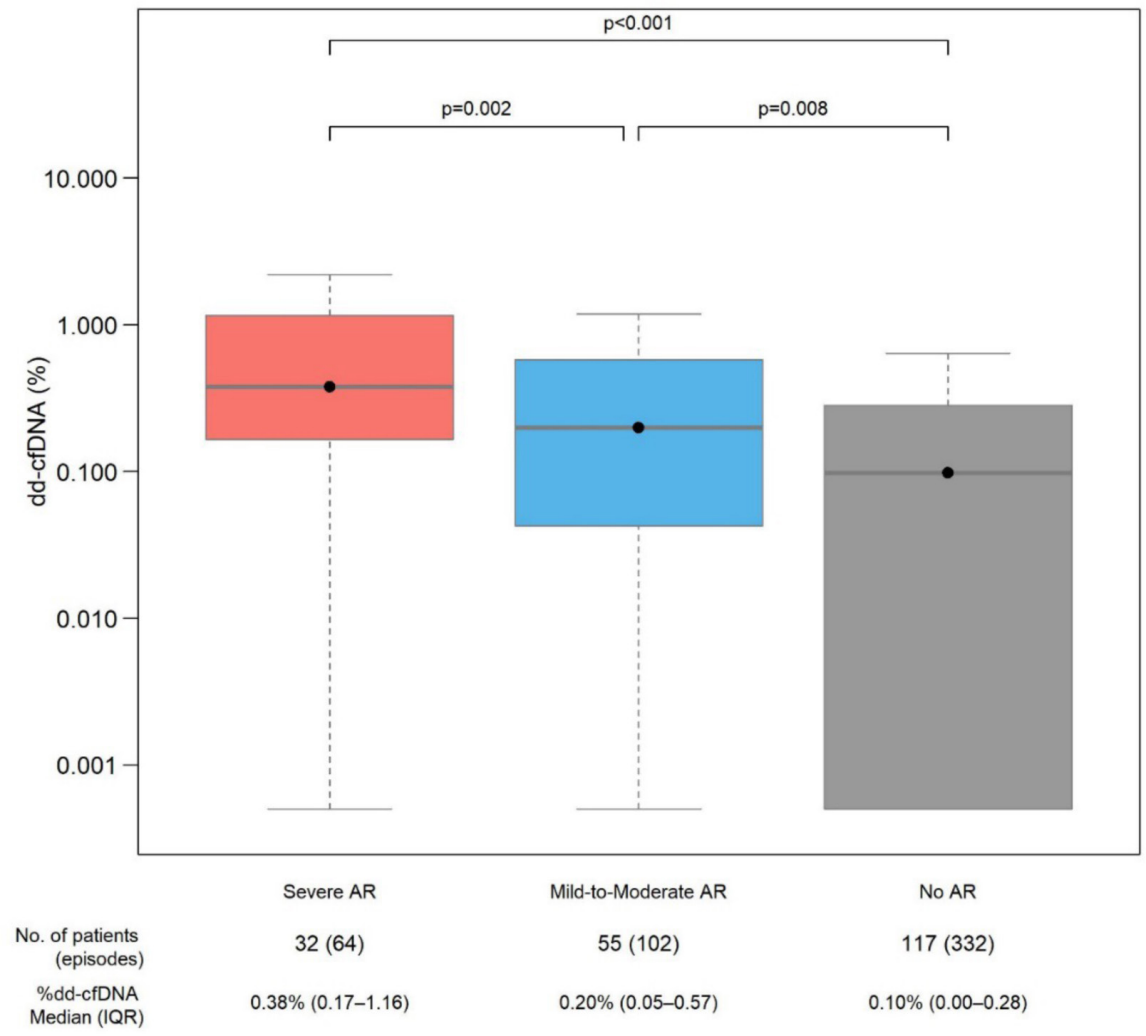
**Percent donor-derived cell-free DNA at the time of acute rejection (AR) diagnosis across rejection severity groups.** Percent donor-derived cell-free DNA (%dd-cfDNA) at the time of AR diagnosis for different AR groups and at matched times for controls with no AR. Severe AR (ACR 3, pAMR grade ≥2, or DSA+/LV dysfunction); mild-to-moderate AR (ACR grade 2 or pAMR grade 1) and controls with no AR, with %dd-cfDNA measured at 2:1 time-matched to the time of AR diagnosis. ACR indicates acute cellular rejection; DSA, donor specific antibodies; LV, left ventricular; and pAMR, patholgic antibody mediated rejection.

Consistent with prior reports, %dd-cfDNA values were elevated in AR and its phenotypes compared with no-rejection controls (Figure 3; Figure S2). Percent dd-cfDNA increased across no AR, mild-to-moderate AR, and severe AR groups. Severe AR showed ≈2-fold higher than mild-to-moderate AR (0.38% versus 0.20%; *P*=0.002) and no AR (0.10%; *P*<0.001), and mild-to-moderate AR also showed a modest increase compared with no AR (*P*=0.008).

When AR was stratified by %dd-cfDNA, risk differed based on %dd-cfDNA levels (Table 3). Among AR patients with an AR episode occurring ≥30 days post-transplant and included in the %dd-cfDNA-stratified analysis (n=55), 26 had %dd-cfDNA <0.15% and 29 had %dd-cfDNA ≥0.15%. In multivariable analyses, AR with %dd-cfDNA ≥0.15% was associated with an increased risk of prolonged LV dysfunction/death (HR, 6.28 [95% CI, 3.04–13.0]; *P*<0.001), death (HR, 4.39 [95% CI, 1.83–10.5]; *P*<0.001), and prolonged LV dysfunction (HR, 9.54 [95% CI, 2.57–35.4]; *P*<0.001). These findings were consistent with the unadjusted analysis (Table 3). Adding %dd-cfDNA categorization to AR-only models improved risk reclassification and discrimination, as assessed by net reclassification improvement and integrated discrimination improvement (Table S2).

**Table 3.**
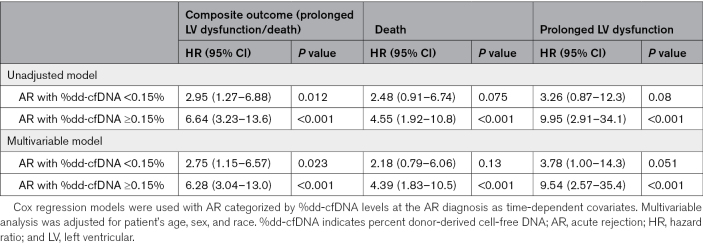
Multivariable Analysis of AR by %dd-cfDNA Categorization With Risk of Adverse Outcomes

In sensitivity analyses (Table S3), using an alternative prespecified %dd-cfDNA cutoff of 0.25% yielded similar associations with poor outcomes: AR with %dd-cfDNA ≥0.25% remained associated with increased risks of the composite outcome (HR, 6.91 [95% CI, 3.24–14.74]; *P*<0.001) and death (HR, 5.74 [95% CI, 2.34–14.10]; *P*<0.001) in multivariable models. In exploratory analyses (Table S4), limited by small stratum sizes (Tables S5 and S6), AR was further stratified by both severity and %dd-cfDNA at diagnosis. In multivariable models, the highest risks were observed in severe AR with %dd-cfDNA ≥0.15% for the composite outcome (HR, 9.00 [95% CI, 3.83–21.1]; *P*<0.001) and death (HR, 8.88 [95% CI, 3.44–22.95]; *P*<0.001). Among patients with mild-to-moderate AR, %dd-cfDNA ≥0.15% was also associated with a higher risk of the composite outcome (HR, 4.02 [95% CI, 1.37–11.81]; *P*=0.011). However, the risk increases in the AR group with %dd-cfDNA <0.15% did not reach statistical significance. Unadjusted models showed similar results (Table S4).

Treatment for AR is summarized in Table S7. In unadjusted descriptive plots of longitudinal %dd-cfDNA relative to the first AR diagnosis (Figure S3), patients who subsequently developed the primary outcome (death or prolonged LV dysfunction; red curve) tended to have higher %dd-cfDNA around the time of AR and showed less decline during post-AR follow-up, whereas patients without these outcomes (blue curve) generally showed decreasing values approaching pre-AR baseline levels.

## Discussion

Despite advances in treatment, AR remains a leading cause of morbidity and mortality following cardiac transplantation.^1,7–9^ Unfortunately, risk stratification remains challenging. In this large multicenter prospective cohort, we demonstrated that AR is associated with a composite outcome of death or prolonged reduction in LVEF and its 2 constituent secondary end points. Furthermore, within AR episodes, elevated %dd-cfDNA at diagnosis (≥0.15%) identified a higher-risk subgroup for adverse outcomes. The patients who go on to develop adverse outcomes show longitudinally elevated %dd-cfDNA at and following diagnosis and despite treatment for AR. We also demonstrated that, in the contemporary era of cardiac transplantation, AR, defined by current ISHLT histological grading,^1^ and EMB-negative AMR additionally defined by DSA+/LV dysfunction,^13^ are associated with death and the composite outcome. Our study established potential %dd-cfDNA thresholds that could be utilized at the bedside to risk stratify patients with AR for poor outcomes.

All patients who developed AR received similar therapies. However, %dd-cfDNA elucidated subgroups that exhibit different allograft injury profiles at diagnosis and after treatment. Those who had %dd-cfDNA levels above a data-derived threshold of 0.15% were at significantly higher risk for adverse outcomes. The prespecified %dd-cfDNA threshold was shown to be predictive of poor outcomes. These high-risk patients also showed longitudinal elevations in %dd-cfDNA after diagnosis and treatment, whereas the low-risk patients showed decreased %dd-cfDNA trends toward the prerejection baseline. The longitudinally elevated %dd-cfDNA could indicate ongoing allograft injury, inadequate treatment response, or a need for additional or different treatment strategies. These patients may benefit from second-line therapies or prolonged treatment duration. The %dd-cfDNA patterns could also be used to select candidates for clinical trials to test new treatment strategies. A biomarker-driven treatment strategy could improve outcomes by targeting therapy to those most at risk while minimizing unnecessary immunosuppression and its associated complications.

In our AR severity subgroup analyses, severe AR was associated with risk of the composite outcome and death. This risk was further stratified by %dd-cfDNA ≥0.15% based on our exploratory analyses. In contrast, mild-to-moderate AR was associated with an increased risk of the composite outcome, but not death. Similarly, in our exploratory analyses, risk stratification by %dd-cfDNA ≥0.15% for mild-to-moderate AR was associated with the composite outcome, but not death. These findings could have been limited by the small sample size of patients with mild-to-moderate AR, successful treatment responses that may have derisked these patients for downstream outcomes, or other unidentified reasons.

The contemporary era in cardiac transplant is marked by rigorous monitoring for early detection and treatment of AR to derisk patients for unfavorable outcomes. This study utilizes %dd-cfDNA as a sensitive, noninvasive biomarker that is commercially available, which is a departure from prior pioneering studies that established the risk association of AR and death using EMB. Assessments of %dd-cfDNA are reproducible, not liable to significant measurement variability,^20^ and sensitive to the different phenotypes of AR, including the recently described category of potentially EMB-negative AMR.^13^ Gene expression profiling is an alternative noninvasive approach used for surveillance in low-risk transplant recipients. It has not been studied for risk assessments in patients with biopsy-confirmed rejection or biopsy-negative forms of rejection.^22–24^ In addition to the use of %dd-cfDNA, this study assessed the association of AR to an intermediate outcome, which is increasingly recognized as a potential harbinger of early death.^7–9^ The study also includes additional forms of rejection, which were not previously assessed.

Our study has several limitations. The sample size was modest, with a minority of patients experiencing AR, particularly in the DSA and LV dysfunction categories. The prevalence of the composite outcome was also low. The sample size and event rates limited our analyses of the risk of poor outcomes in AR stratified by %dd-cfDNA and severity to being exploratory in nature. Our limited sample size also precluded the stratification of AMR or ACR by %dd-cfDNA at diagnosis. In addition, there was likely interobserver variability in EMB pathological assessments within the 5 GRAfT centers that is unaccounted for in our analyses. The %dd-cfDNA thresholds proposed were either data-derived based on this cohort or reported in prior studies^13,18^ but differ from the thresholds used in other cohort studies.^25,26^ Furthermore, our multivariable regressions adjusted only for age, sex, and race to avoid overfitting the data given the low event rate. These %dd-cfDNA risk stratification models can be used in conjunction with standard risk assessment tools. We also did not include coronary artery vasculopathy as a secondary end point due to insufficient data; this would have been a useful intermediate outcome to describe allograft dysfunction. In addition, the longitudinal trends in %dd-cfDNA are only descriptive in nature and warrant further exploration into possible prognostic utility. The study did not evaluate the effect of treatment on the effectiveness of treating AR and on the risk of poor outcomes. Future studies with larger sample sizes may validate these %dd-cfDNA thresholds and address the other limitations. Future studies should assess whether prolonged treatment in high-risk patients or other approaches could reduce the risk of these adverse outcomes. Future studies should also assess the reversibility of the intermediate outcome used in this study and assess the generalizability of these findings in other cohorts. Our findings should be viewed as hypothesis-generating, pending further assessments in future studies.

In conclusion, in this contemporary era with new definitions and rigorous patient monitoring, AR remains a risk factor for both death and an intermediate end point. Our findings suggest that risk remains significant in patients with AR, particularly in those with elevated %dd-cfDNA at diagnosis. These patients showed longitudinally elevated %dd-cfDNA after treatment. Our findings suggest that %dd-cfDNA could be a promising noninvasive biomarker, enabling the monitoring of treatment response and risk stratification for mortality. Pending validation studies, these findings support the integration of %dd-cfDNA into clinical risk decision-making in heart transplant recipients.

## Article Information

### Disclosures

None.

### Supplemental Material

Tables S1–S7

Figures S1–S3

## Supplementary Material


