# Supplementary material for: Donor-Derived Cell-Free DNA at Diagnosis of Cardiac Allograft Rejection Stratifies Risk of Mortality and Graft Dysfunction
Source: Circ Heart Fail. 2026 Jun 15;19(7):e013718. doi: 10.1161/CIRCHEARTFAILURE.125.013718 (PMC13390989; doi:10.1161/CIRCHEARTFAILURE.125.013718)
Supplement: Supplementary file 1 [file hhf-19-e013718-s001.pdf]

SUPPLEMNTAL MATERIAL

Supplementary Figures

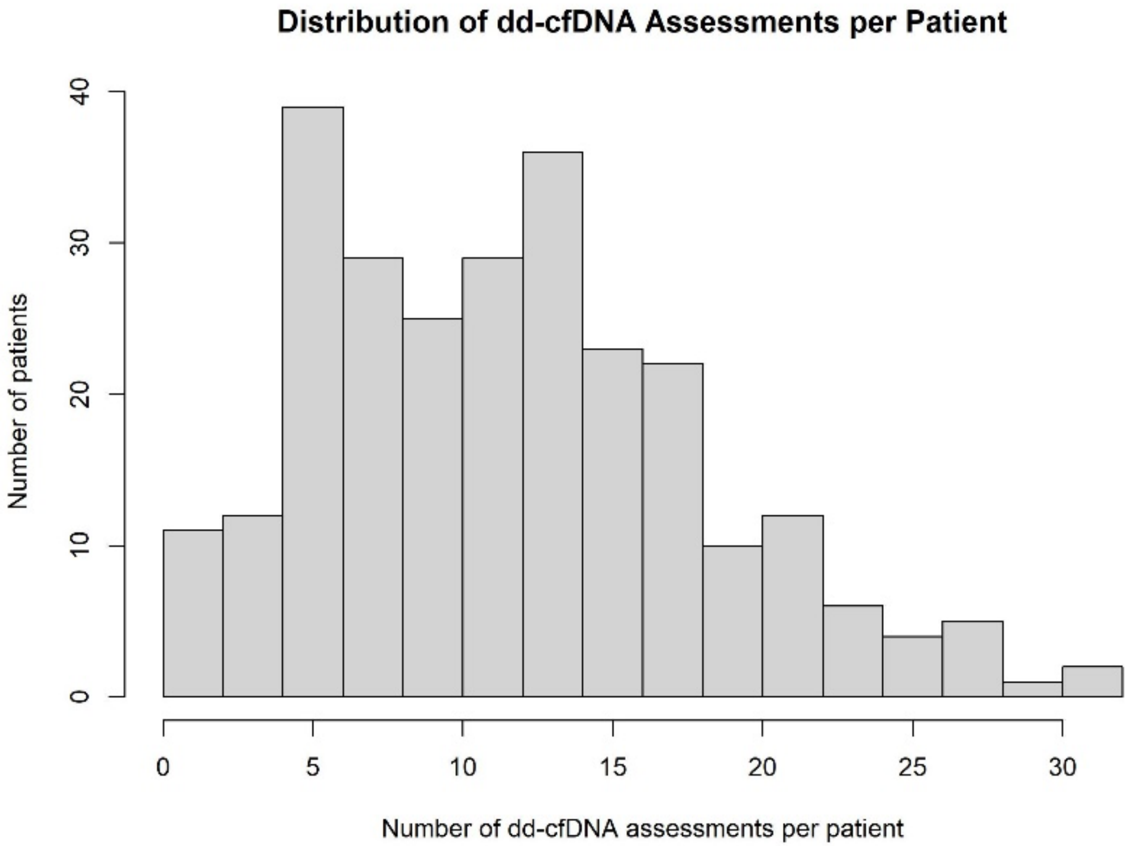

Figure S1: Distribution of Donor-Derived Cell-Free DNA Assessments per Patient. A Histogram of dd-cfDNA assessments per patient during the follow-up period is shown.

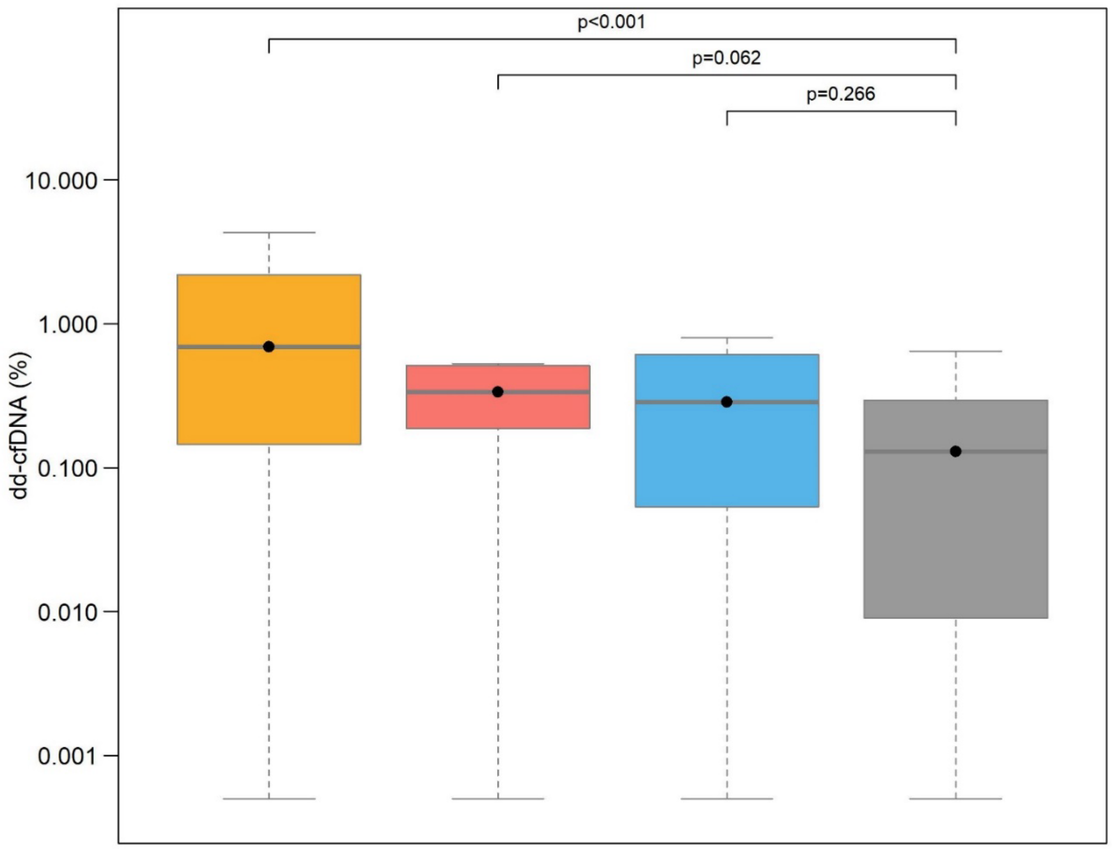

	pAMR+: Grade 2+	DSA+/LV Dysfunction	pAMR+: Grade 1	no DSA/LV Dysfunction / pAMR-
No. of patients (episodes)	12 (31)	22 (27)	18 (48)	125 (318)
%dd-cfDNA Median (IQR)	0.69% (0.15–2.19)	0.34% (0.22–0.51)	0.29% (0.06–0.61)	0.13% (0.01–0.30)

Figure S2a: Percent Donor-Derived Cell-Free DNA at the Time of AMR Diagnosis Across pAMR Grade and EMB-negative AMR. Donor-derived cell-free DNA (%) at the time of AMR diagnosis for pAMR grades 1-2, EMB-negative AMR and controls with no AMR, with %dd-cfDNA measured at 2:1 time-matched to the time of AMR diagnosis.

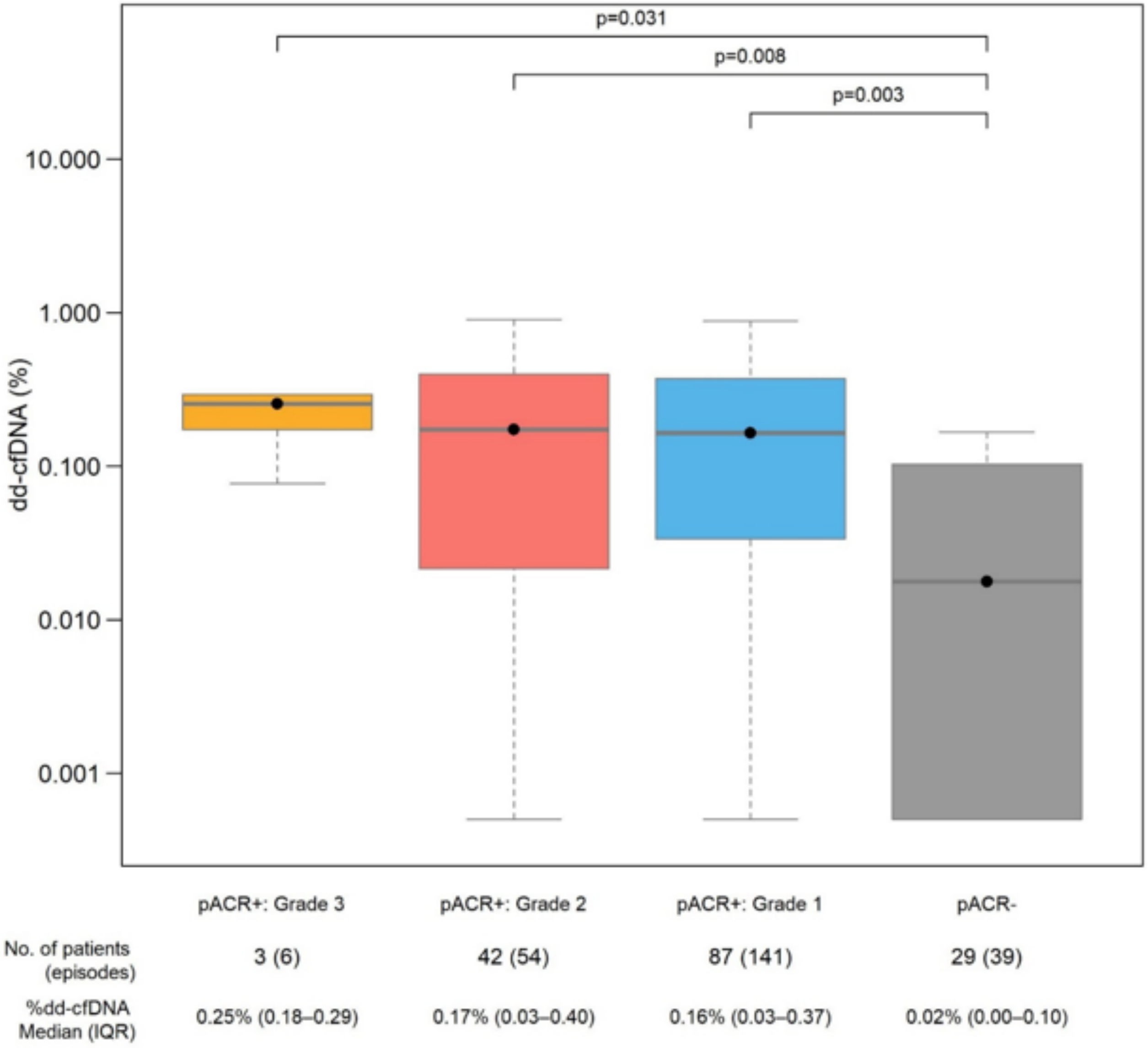

Figure S2b: Percent Donor-Derived Cell-Free DNA at the Time of ACR Diagnosis Across ACR Grade. Donor-derived cell-free DNA (%) at the time of ACR diagnosis for ACR grades 1-3, and controls with no ACR, with %dd-cfDNA measured at 2:1 time-matched to the time of ACR diagnosis.

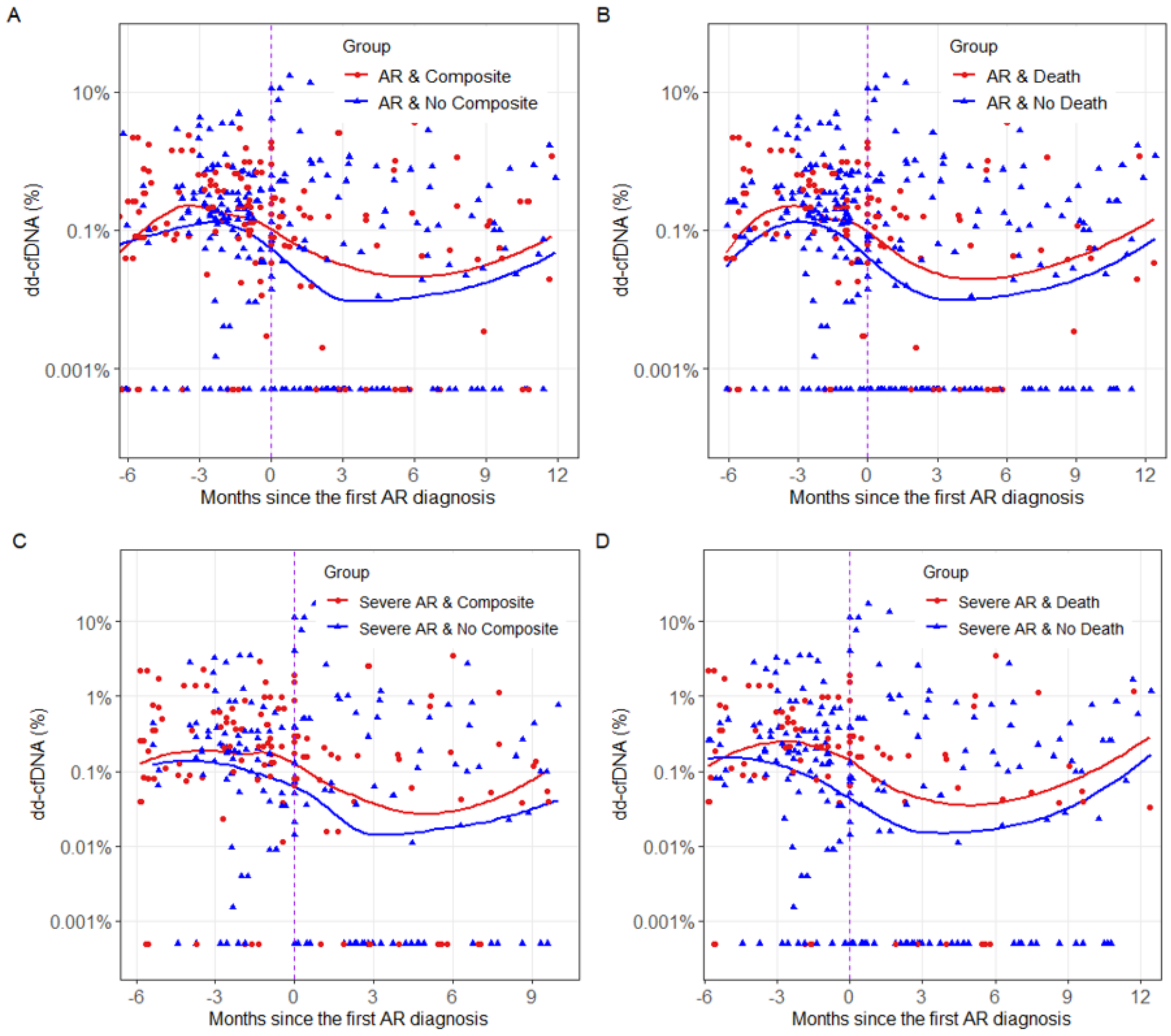

Figure S3: Trend of longitudinal %dd-cfDNA measurement relative to the first AR diagnosis. Loess smoothed curves are shown with or without Prolonged LV Dysfunction /death, respectively. (A-B) included all AR; (C-D) included Severe AR.

Supplementary Tables

Table S1. Analyses of Risk for Death and Prolonged LV Dysfunction with AR by severity

	Composite outcome (prolonged LV dysfunction/death)		Death		Prolonged LV Dysfunction	
	HR (95% CI)	p	HR (95% CI)	p	HR (95% CI)	P
**Univariate model with AR severity***						
Mild-to-moderate	3.31 (1.53, 7.17)	0.002	2.13 (0.78, 5.79)	0.14	5.17 (1.69, 15.8)	0.004
Severe	6.69 (3.14, 14.3)	<0.001	5.60 (2.37, 13.2)	<0.001	5.29 (1.11, 25.2)	0.037
**Multivariable model with AR severity***						
Mild-to-moderate	3.11 (1.40, 6.90)	0.005	2.05 (0.74, 5.66)	0.17	5.56 (1.77, 17.4)	0.003
Severe	6.30 (2.91, 13.6)	<0.001	4.82 (2.00, 11.6)	<0.001	4.78 (0.96, 23.8)	0.057
**Unadjusted model with severe AR determined by biopsy**						
Severe (ACR grade ≥3, pAMR grade ≥2)	7.72 (2.68, 22.3)	<0.001	7.80 (2.28, 26.7)	0.001	8.68 (1.10, 68.4)	0.04
**Multivariable model with severe AR determined by biopsy**						
Severe AR (ACR grade ≥3, pAMR grade ≥2)	6.59 (2.17, 20.0)	<0.001	6.91 (1.93, 24.8)	0.003	5.55 (0.68, 45.6)	0.111

Note: *AR severity was determined as mild-to-moderate (ACR grade 2 or pAMR grade 1) and severe (ACR grade 3, pAMR grade ≥2, or DSA+/LV dysfunction [donor-specific antibody presence and LVEF drop ≥10% to a value≤50%]). Multivariable Cox regression analysis was adjusted for patient's age, sex, and race.

Table S2. Net Reclassification Improvement (NRI) and Integrated Discrimination Improvement (IDI) for comparing the model of AR adding %dd-cfDNA categorization and the AR-based model

	Composite outcome (prolonged LV dysfunction /death)		Death		Prolonged LV Dysfunction	
	NRI	IDI	NRI	IDI	NRI	IDI
**Unadjusted models**						
Model 1: AR<br>Model 2: AR and %dd-cfDNA categorization	0.0546	0.0221	0.0946	0.0065	-0.0913	0.0314
**Multivariable models**						
Model 1: AR<br>Model 2: AR and %dd-cfDNA categorization	0.0607	0.0274	0.1008	0.0124	0.5300	0.0155

Note: NRI and IDI were used to measure the incremental model-performance to compare the new model (model 2) of adding %ddcfDNA to the AR categorization and the reference model (model 1) using only AR diagnosis. These measures were calculated for each time-to-event outcome at a 2-year time window based on the unadjusted Cox models (with AR as covariate) in Table 2 and multivariable models (with AR and %dd-cfDNA categorization as covariates) in Table 3.

Table S3. Analysis of AR with %dd-cfDNA Dichotomized at 0.25% at the Time of AR Diagnosis and Risk of Adverse Outcomes

	Composite outcome (prolonged LV dysfunction /death)		Death		Prolonged LV Dysfunction	
	HR (95% CI)	p	HR (95% CI)	p	HR (95% CI)	p
**Unadjusted models**						
AR with ddcfDNA<0.25%	3.27 (1.51, 7.08)	0.003	2.46 (0.97, 6.27)	0.059	4.04 (1.22, 13.4)	0.022
AR with ddcfDNA≥0.25%	6.87 (3.22, 14.7)	<0.001	5.19 (2.12, 12.7)	<0.001	8.82 (2.27, 34.3)	0.002
**Multivariable models**						
AR with ddcfDNA<0.25%	2.97 (1.35, 6.53)	0.007	2.04 (0.79, 5.27)	0.143	4.36 (1.29, 14.7)	0.018
AR with ddcfDNA≥0.25%	6.91 (3.24, 14.7)	<0.001	5.74 (2.34, 14.1)	<0.001	7.92 (1.88, 33.4)	0.005

Note: Cox regression models were used with time-dependent covariates for AR with %dd-cfDNA dichotomized at 0.25% at diagnosis. Multivariable analysis was adjusted for patient's age, sex, race.

Table S4. Analysis of AR by Severity and %dd-cfDNA Levels Dichotomized at 0.15% with Risk of Adverse Outcomes

	Composite outcome (prolonged LV dysfunction /death)		Death		Prolonged LV Dysfunction	
	HR (95% CI)	p	HR (95% CI)	p	HR (95% CI)	p
**Unadjusted model for AR severity and %dd-cfDNA categorization**						
Mild-to-moderate with ddcfDNA<.15%	2.89 (1.10, 7.59)	0.032	2.91 (0.98, 8.67)	0.055	2.86 (0.61, 13.4)	0.18
Mild-to-moderate with ddcfDNA≥.15%	4.15 (1.42, 12.1)	0.009	1.02 (0.14, 7.73)	0.98	11.87 (3.07, 45.8)	<0.001
Severe with ddcfDNA<.15%	3.12 (0.74, 13.3)	0.122	1.51 (0.20, 11.4)	0.69	4.52 (0.56, 36.2)	0.16
Severe with ddcfDNA≥.15%	9.79 (4.22, 22.8)	<0.001	9.57 (3.79, 24.1)	<0.001	6.63 (0.80, 55.2)	0.08
**Multivariable model for AR severity and %dd-cfDNA categorization**						
Mild-to-moderate with ddcfDNA<.15%	2.65 (0.95, 7.40)	0.064	2.78 (0.89, 8.69)	0.079	3.04 (0.60, 15.4)	0.18
Mild-to-moderate with ddcfDNA≥.15%	4.02 (1.37, 11.8)	0.011	1.01 (0.13, 7.66)	0.993	11.1 (2.69, 45.5)	<0.001
Severe with ddcfDNA<.15%	2.96 (0.67, 13.1)	0.153	1.15 (0.15, 8.84)	0.891	5.05 (0.57, 44.9)	0.147
Severe with ddcfDNA≥.15%	9.00 (3.83, 21.1)	<0.001	8.88 (3.44, 22.9)	<0.001	5.31 (0.57, 49.1)	0.141

Note: Cox regression models were used with time-dependent covariates for AR by severity and %dd-cfDNA at the AR diagnosis. AR severity was determined as mild-to-moderate (ACR grade 2 or pAMR grade 1) and severe (ACR grade 3, pAMR grade ≥2, or DSA+/LV dysfunction [donor-specific antibody presence and LVEF drop ≥10% to a value≤50%]). Multivariable analysis was adjusted for patient's age, sex, and race.

Table S5. Definitions and counts of rejection phenotypes and severity categories included in the %dd-cfDNA-stratified Cox analyses

**Rejection Type**	**Severity**	**Definition**	Patients (n)
**AR**	**Mild-to-Moderate**		**32**
	**Severe**		**23**
pAMR	Mild-to-Moderate	Grade = 1	9
	Severe	Grade ≥2	5
ACR	Mild-to-Moderate	Grade = 2	23
	Severe	Grade = 3	2
DSA+/LV dysfunction	Severe	donor-specific antibody presence and LVEF drop ≥10% to a value≤50%	16
		**Total**	55

Table S6: Number of Patients in AR Category Stratified by %dd-cfDNA Threshold

AR Severity	%dd-cfDNA ≥ 0.15	%dd-cfDNA < 0.15
Mild-to-moderate AR	18	14
Severe AR	11	12

Table S7. Treatment for ACR and AMR by GRAfT Center

Center	Acute Cellular Rejection	Antibody Mediated Rejection
Center 1	IV steroids with hemodynamic instability. Oral steroids for others.	IV steroids Plex/Rituximab/IVIG
Center 2	IV steroids, ATG with hemodynamic instability	IV steroids Plex/IVIG/Bortezomib
Center 3	Oral steroids or IV steroid + ATG with low EF or hemodynamic instability	IV steroids Plex/IVIG/Thymo +Ritux or Bortezomib
Center 4	Oral steroids or IV steroid + ATG with low EF or hemodynamic instability	IV steroids Plex/IVIG/Ritux and/or Bortezomib
Center 5	Oral steroids or IV steroid + ATG with low EF or hemodynamic instability	IV steroids Plex/IVIG

Note: IV=intravenous, Plex=plasmapheresis, IVIG=Intravenous immunoglobulin, Thymo-thymoglobulin, Ritux=Rituximab
